# Polymorphisme de *Plasmodium falciparum* et mutations des gènes de résistance *Pfcrt* et *Pfmdr1* dans la zone de Nanoro, Burkina Faso

**DOI:** 10.11604/pamj.2021.39.118.26959

**Published:** 2021-06-10

**Authors:** Paul Sondo, Biebo Bihoun, Bérenger Kabore, Marc Christian Tahita, Karim Derra, Toussaint Rouamba, Seydou Nakanabo Diallo, Adama Kazienga, Hamidou Ilboudo, Innocent Valea, Zekiba Tarnagda, Hermann Sorgho, Thierry Lefevre, Halidou Tinto

**Affiliations:** 1 Institut de Recherche en Sciences de la Santé, Unité de Recherche Clinique de Nanoro (IRSS-URCN), Bobo-Dioulasso, Burkina Faso,; 2 Institut National de Santé Publique, Centre Muraz de Bobo-Dioulasso, Bobo-Dioulasso, Burkina Faso,; 3 Laboratoire Mixte International sur les Vecteurs (LAMIVECT), Bobo Dioulasso, Burkina Faso,; 4 Maladies Infectieuses et Vecteurs, Ecologie, Génétique, Evolution et Contrôle (MIVEGEC), Université de Montpellier, Institut de Recherche pour le Développement (IRD), Centre National pour la Recherche Scientifique (CNRS), Montpellier, France,; 5 Centre de Recherche en Écologie et Évolution de la Santé (CREES), Montpellier, France

**Keywords:** Plasmodium falciparum, msp1, msp2, Pfcrt, Pfmdr1, Plasmodium falciparum, msp1, msp2, Pfcrt, Pfmdr1

## Abstract

**Introduction:**

sur le plan génétique, Plasmodium falciparum(P. falciparum) est une espèce extrêmement polymorphe. Il existe une diversité de souches parasitaires qui infestent les individus vivant en zone d´endémie palustre. La présente étude vise à étudier la relation entre le polymorphisme de P. falciparum et les mutations au niveau des gènes Pfcrt et Pfmdr1 dans la zone de Nanoro au Burkina Faso.

**Méthodes:**

les échantillons sanguins de porteurs de plasmodiums résidant dans le district sanitaire de Nanoro ont fait l´objet d´un génotypage par PCR nichée. Les mutations au niveau des gènes de résistance du parasite aux antipaludiques ont été détectées par la technique PCR-RFLP.

**Résultats:**

les échantillons de 672 patients ont été génotypés avec succès. Aucune famille allélique des gènes msp1et msp2n´avaient une susceptibilité accrue à développer des mutations au niveau des gènes de résistance. Par contre, les souches mutantes de ces gènes étaient significativement plus importantes dans les infections monoclonales que dans les infections multi clonales.

**Conclusion:**

cette étude fournit un aperçu global de la relation entre le polymorphisme de P. falciparum et les mutations au niveau des gènes de résistance. Ces données contribueront sans doute à améliorer les connaissances sur la biologie du parasite et de ses mécanismes de résistance aux antipaludiques.

## Abstract (en)

**Introduction:**

from a genetic point of view P. falciparumis extremely polymorphic. There is a variety of parasite strains infesting individuals living in malaria endemic areas. The purpose of this study is to investigate the relationship between polymorphisms in Plasmodium falciparum parasites and Pfcrt and Pfmdr1 gene mutations in Nanoro area, Burkina Faso.

**Methods:**

blood samples from plasmodium carriers residing in the Nanoro Health District were genotyped using nested PCR. Parasite gene mutations associated with resistance to antimalarial drugs were detected by PCR-RFLP.

**Results:**

samples of 672 patients were successfully genotyped. No msp1and msp2allelic families exhibited an increase in developing mutations in resistance genes. However, mutant strains of these genes were present at greater levels in monoclonal infections than in multi-clonal infections.

**Conclusion:**

this study provides an overview of the relationship between polymorphisms in Plasmodium falciparum parasites and mutations in resistance genes. These data will undoubtedly contribute to improving knowledge of the parasite´s biology and its mechanisms of resistance to antimalarial drugs.

## Introduction

Le paludisme demeure une préoccupation sanitaire mondiale avec 228 millions de cas et 405.000 décès rapportés dans le monde en 2018 [1]. Au Burkina Faso, le nombre de cas était estimé à 7.875.575 dont 12.725 décès en 2018 [1]. De tous les hématozoaires pouvant parasiter l´homme, l´espèce *P. falciparum* est la plus dangereuse du fait de sa forte implication dans la mortalité attribuable au paludisme et dans la résistance aux antipaludiques.

Sur le plan génétique, *P. falciparum* est une espèce extrêmement polymorphe. Il existe une grande diversité de souches parasitaires qui infestent les individus vivant en zone d´endémie palustre [2-4]. Malgré l´existence de plusieurs marqueurs de polymorphisme, les protéines de surface du mérozoïte (*msp1* et *msp2*) sont les plus appropriés, du fait de leur implication dans l´invasion du globule rouge par le mérozoïte. Le gène de la *msp1* est situé sur le chromosome 9 et les variations au niveau des séquences répétées de son block 2 permettent de subdiviser les populations parasitaires en trois familles alléliques K1, MAD20 et RO33 [5,6]. Le gène de la *msp2* est situé sur le chromosome 2 et le polymorphisme de son block central subdivise les populations parasitaires en deux familles alléliques 3D7 et FC27. Ces marqueurs de polymorphisme sont en effet les plus couramment utilisés pour distinguer les populations parasitaires [6].

Parallèlement, la résistance de *P. falciparum* aux antipaludiques constitue un grand obstacle à la lutte contre le paludisme dans la plupart des pays endémiques. Face à la résistance à la Chloroquine (CQ), les CTA ont été recommandées par l´OMS pour le traitement du paludisme simple. Le Burkina Faso a adopté les combinaisons Artésunate-Amodiaquine (ASAQ), Artéméther-Luméfantrine (AL) et Dihydro-artémisinine-Pipéraquine (DHAPPQ) pour la prise en charge du paludisme simple [7,8]. Il a été clairement démontré que des mutations au niveau des gènes *Pfmdr1* et *Pfcrt* étaient associées à la résistance aux amino-4-quinoléines. Le gène *Pfcrt* est localisé sur le chromosome 7 et code pour une protéine membranaire impliquée dans le transport du médicament vers l´intérieur de la cellule. Une mutation au niveau du codon 76 qui induit un changement au niveau de la protéine par remplacement de l´acide aminé lysine (K) par la thréonine (T) est fortement associée à la résistance à la Chloroquine (CQ) et à l´Amodiaquine par résistance croisée [9,10]. Le gène *Pfmdr1* est localisé sur le chromosome 5 et code pour une protéine membranaire dont l´implication dans la résistance à la Chloroquine et à l´Amodiaquine a été évoquée. Plusieurs points de mutations y sont décrits dont le point N86Y [11,12].

Cependant, la question de savoir s´il existe des associations entre certaines variantes génétiques du parasite et les mutations au niveau des gènes de résistances reste encore non clairement élucidée. La présente étude vise à étudier la relation entre le polymorphisme de *P. falciparum* et les mutations au niveau des gènes *Pfcrt* et *Pfmdr1* dans la zone de Nanoro au Burkina Faso.

## Méthodes

**Site de l´étude**: l´étude s´est déroulée à Nanoro dans la Province du Boulkiemdé au Burkina Faso en Afrique de l´Ouest. Le District sanitaire de Nanoro (DSN) est l´un des cinq districts de la région sanitaire du Centre-Ouest du Burkina Faso. La population totale de la zone était estimée à 180.357 habitants en 2019 et couvre une superficie de 1302 Km^2^, soit 5,98% de la superficie de la région du centre ouest [13]. Les études entomologiques les plus récentes conduites au Burkina Faso indiquent que les vecteurs les plus courants sont *Anopheles gambiae s.s*. et *An. arabiensis. P. falciparum* est le principal agent pathogène, suivi de *P. malariae* et de *P. ovale* [14].

**Provenance des échantillons**: les échantillons de sang séchés sur papier Whatman (confettis) utilisés pour cette étude ont été collectés chez des patients souffrant de paludisme à *P. falciparum* et enrôlés dans l´étude Pharmacovigilance des combinaisons thérapeutique à base d´Artémisinine en Afrique [15,16]. Toutefois, seuls les échantillons collectés au Jour 0, avant l´administration du traitement ont été utilisé pour cette investigation.

**Analyse moléculaires**: l´ADN parasitaire a été extrait des confettis en utilisant les Kits QIamp DNA miniKit (Qiagen, Germany) en suivants les instructions du fabricant. Le génotypage du bloc 2 de *msp1* et du bloc 3 de *msp2* s´est fait par la technique de la PCR nichée selon un protocole décrit antérieurement [3,17]. Ensuite, l´amplification des gènes *Pfcrt* et *Pfmdr1* s´est fait par PCR nichée suivi d´une digestion des produits PCR par les enzymes de restriction. L´enzyme de restriction Apo I (NEB) a été utilisé pour le point *Pfcrt K76T* et *Afl III* (NEB) a été utilisé pour le point *Pfmdr1* N86Y [18]. Les fragments d´ADN ont été séparés par électrophorèse sur gel d´agarose contenant du Bromure d´Ethidium. La révélation des fragments s´est faite au moyen d´un Trans illuminateur Ultraviolet.

**Analyse des données**: les résultats des analyses moléculaires ont été saisis sur un fichier Excel. Les données ont ensuite été analysées avec le logiciel R [19]. Un échantillon était considéré comme appartenant à une famille allélique donnée s´il y avait l´apparition d´au moins une bande suite à l´amplification de l´ADN de cet échantillon avec les amorces spécifiques de la famille en question. En cas d´absence de bande, l´échantillon était considéré comme n´étant pas de cette famille allélique. Les génotypes de *Pfcrt* et *Pfmdr1* ont été déterminés sur la base de la présence ou l´absence de l´allèle sauvage/mutant au niveau des *loci*. Le test de Chi-carrée était utilisé pour comparer les proportions avec un seuil de significativité de 5%.

**Considérations éthiques**: tous les participants ont donné leur consentement libre et éclairé avant l´inclusion dans l´étude. L´étude a été conduite en respectant les principes de bonnes pratiques cliniques et la règlementation en vigueur en matière d´essai clinique au Burkina Faso. Le protocole de l´étude a été approuvé par le comité d´éthique institutionnelle du Centre Muraz et par le comité d´éthique pour la recherche en Santé du Ministère de la Santé du Burkina Faso.

## Résultats

Au total, 680 patients ont été inclus dans l´étude d´évaluation de l´efficacité thérapeutique d´ASAQ comparée à AL. Tous les échantillons disponibles (n=676) ont été considérés de façon systématique pour cette investigation (indépendamment de l´issu du traitement avec ASAQ ou AL). Les échantillons de 672 (99,4%) participants ont été génotypés avec succès pour *msp1* et *msp2* et pour *Pfcrt* et *Pfmdr1*. La prévalence de la mutation *Pfcrt* K76T était de 20,6% (120/581) et celle de *Pfmdr1* N86Y était de 08,1% (49/604). Les échantillons portant des infections mixtes avec à la fois l´allèle sauvage et l´allèle mutant étaient au nombre de 91 (13,5%) pour *Pfcrt* et de 68 (10,1%) pour *Pfmdr1*.

**Figure 1:**
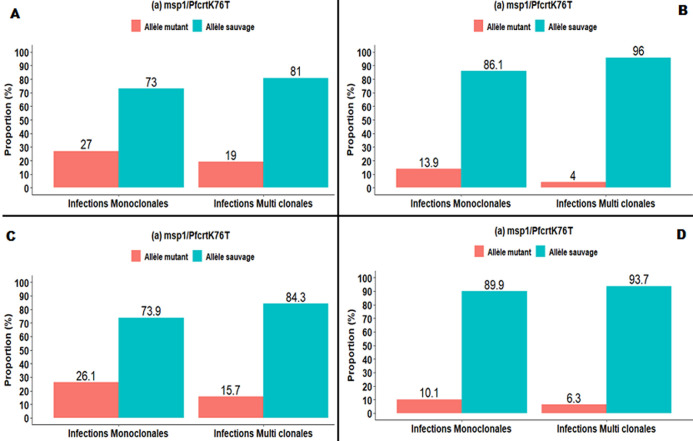
prévalence des souches sauvages et mutantes de *Pfcrt* et *Pfmdr1* au niveau des infections monoclonales versus infections polyclonales; la couleur rouge représente les souches mutantes et la couleur bleue représente les souches sauvages

Le Tableau 1 illustre la relation entre la diversité génétique de *P. falciparum* et les mutations au niveau du gène *Pfcrt*. Aucune différence statistiquement significative de la présence de l´allèle sauvage ou mutant du gène *Pfcrt* selon le profile polymorphique des gènes *msp1* n´a été observée (Tableau 1). Par contre, la prévalence de l´allèle mutant du gène *Pfcrt* était significativement plus élevée dans les infections monoclonales comparées aux infections polyclonales avec *msp1* (χ^2^=12,02 p=0,001, Figure 1 A) et *msp2* (χ^2^=17,91 p<0,001, Figure 1 C) respectivement. Le Tableau 1 illustre la relation entre le polymorphisme de *msp2* et la mutation K76T du gène *Pfcrt*. Aucune variation significative de la prévalence de l´allèle mutant ou de l´allèle sauvage de *Pfcrt* entre familles allélique de *msp2* n´a été observée (Tableau 2). La relation entre la variabilité allélique de *msp1* et la mutation *Pfmdr1* N86Y est représentée dans le Tableau 3.

**Tableau 1:** relation entre la variabilité allélique de *msp1* et la mutation *Pfcrt* K76T

Profile polymorphique de msp1	K1N=142	MAD20 N=52	RO33N=50	K1MAD20N=94	K1-RO33N=103	RO33MAD20N=16	K1-MAD20-RO33 N=124
*Pfcrt* K76 n(%)	101(71,1)	37(71,1)	40(80,0)	76(80,8)	81(78,6)	16(100)	110(88,7)
*Pfcrt* T76 n(%)	41(28,9)	15(28,9)	10(20,0)	18(19,2	22(21,4)	0(0)	14(11,3

**Tableau 2:** relation entre la variabilité allélique de *msp2* et la mutation *Pfcrt* K76T

Profile polymorphique de *msp2*	3D7 N=191	FC27N=85	3D7-FC27 N=305
*Pfcrt* K76 (n (%))	140(73,3)	64(75,3)	257(84,3)
*Pfcrt* T76 (n (%))	51(26,7)	21(24,7)	48(15,7)

**Tableau 3:** relation entre la variabilité allélique de *msp1* et la mutation *Pfmdr1* N86Y

Profile polymorphique de *msp1*	K1 N=141	MAD20 N=56	RO33 N=54	K1MAD20 N=110	K1-RO33 N=100	RO33MAD20 N=19	K1-MAD20-RO33 N=124
*Pfmdr1* N86 n(%)	125(88,6)	44(78,6)	47(87,0)	107(97,3)	93(93,0)	18(94,7)	121(97,6)
*Pfmdr1* Y86 n(%)	16(11,4)	12(21,4)	7(13,0)	3(2,7)	7(7,0)	1(5,3)	3(2,4)

Aucune famille allélique de *msp1* n´était spécifiquement associée à la présence de mutation *Pfmdr*1 N86Y. Tout comme au niveau du gène *Pfcrt*, la fréquence de l´allèle mutant 86Y était plus élevée dans les infections monoclonales que dans les infections polyclonales avec *msp1* (χ^2^=19,73 p<0,0001, Figure 1 B) et *msp2* (χ^2^=9,72 p=0,002, Figure 1 D) respectivement.

Le Tableau 4 présente la relation entre famille allélique de *msp2* et la mutation *Pfmdr1* N86Y. A ce niveau également, aucune famille allélique de *msp2* n´était spécifiquement associée à la présence de mutation *Pfmdr1* Y184F (Tableau 4).

**Tableau 4:** relation entre la variabilité allélique de *msp2* de *P. falciparum* et la mutation *Pfmdr1* N86Y

Profile polymorphique de *msp2*	3D7 N=196	FC27 N=90	3D7-FC27 N=318
*Pfmdr1* N86 n(%)	180(91,8)	77(85,6)	298(93,7)
*Pfmdr1* Y86 n(%)	16(8,2)	13(14,4)	20(6,3)

## Discussion

La relation entre la diversité génétique de *P. falciparum* et la résistance aux antipaludéens est cruciale mais reste jusque-là un phénomène non clairement élucidé. Dans cette étude nous avons exploré la susceptibilité de certaines variantes de *msp1* et de *msp2* à développer des mutations au niveau des gènes *Pfcrt* et *Pfmdr1*. En effet, les mutations aux niveaux de ces gènes représentent une sorte de mécanismes d´adaptation parasitaire pour survire dans les conditions environnementales défavorables en l´occurrence la pression médicamenteuse. Par exemple, l´utilisation de la chloroquine pour le traitement du paludisme avait entrainé une forte prévalence des souches mutantes au Burkina Faso. Son remplacement par les CTA dans les années 2005 s´est accompagné d´une diminution drastique de la prévalence de ces souches mutantes avec un retour progressif des souches sauvages de *Pfcrt* et *Pfmdr1* qui sont chloroquino sensibles [20].

Dans l´ensemble, aucune famille allélique de *msp1* ou de *msp2* n´était spécifiquement associée à une susceptibilité accrue de porter des souches mutantes de *Pfcrt* ou de *Pfmdr1*. Cela voudrait dire que les facteurs favorisants l´apparition de ces mutations dont la pression médicamenteuse affectent toutes les variantes de *msp1* et de *msp2* de la même manière. Ce résultat était attendu, puisque l´inversion du profile des souches en termes de résistance induite par le changement de la politique de traitement ne s´est pas accompagné d´un changement de distribution des profils polymorphiques de *msp1* et de *msp2*. Avant ou après le changement de la politique de traitement, la même distribution a été toujours observée avec une prédominance de la famille allélique K1, suivie de MAD20 et de RO33 pour *msp1*. Pour *msp2*, la famille allélique 3D7 a toujours été dominant par rapport à FC27 au Burkina Faso [4,21].

Nos résultats montrent également que les souches mutantes étaient significativement plus importantes dans les infections monoclonales que dans les infections multi-clonales. Nous postulons que cela résulte de la compétition entre souches génétiquement différentes du parasite au sein de l´hôte et cette compétition serait en défaveur des souches mutantes [2]. Cette compétition entre souches distinctes avec effet suppresseur pour les souches mutantes avait aussi été observée dans le modèle animal [22]. En effet, le fait que les souches mutantes soient moins compétitives pourrait s´expliquer par le coût de cette mutation conférant la résistance [23,24]. Il a été démontré que l´aptitude des parasites à survivre en l´absence de traitement se réduisait lorsqu´ils développaient des mutations conférant la résistance comparativement aux souches sauvages [23,24]. La distinction des souches parasitaires par gel d´agarose dans le cadre de cette étude ne permettait pas de déterminer la contribution de chaque génotype parasitaire dans les infections mixtes, ce qui est une limite de notre étude. Par conséquent, le constat en rapport avec la disparition des souches mutantes dans les infections multi-clonales mérite des investigations plus poussées visant à déterminer avec précision les facteurs défavorisant.

## Conclusion

Cette étude fournit un aperçu global de la relation entre le polymorphisme de *P. falciparum* et les mutations au niveau des gènes de résistance *Pfcrt* et *Pfmdr1*. Aucune famille allélique des gènes *msp1* et *msp2* n´avait une susceptibilité accrue à développer des mutations au niveau des gènes de résistance. Par contre les souches mutantes étaient significativement plus importantes dans les infections monoclonales que dans les infections multi clonales. Ces données contribueront sans doute à améliorer les connaissances sur la biologie du parasite.

### Etat des connaissances sur le sujet

- P. falciparum est une espèce extrêmement polymorphe;
- Les gènes des protéines de surface du mérozoïte (msp1 et msp2) sont couramment utilisés pour distinguer les souches parasitaires;
- Les mutations au niveau des gènes Pfmdr1 et Pfcrt sont associées à la résistance aux amino-4-quinoléines.

### Contribution de notre étude à la connaissance

- Aucune famille allélique des gènes msp1 et msp2 n´avait une susceptibilité accrue à développer des mutations au niveau des gènes de résistance;
- Les souches mutantes des gènes de résistance Pfcrt et Pfmdr1 étaient significativement plus importantes dans les infections monoclonales que dans les infections multi clonales.

## Remerciements

Nous remercions tous les participants de l´étude et toute la communauté du district sanitaire de Nanoro. Nous remercions le personnel de l´Unité de Recherche Clinique de Nanoro (URCN) et des Centres de Santé et de Promotion Sociales (CSPS) de Nanoro et de Nazoanga.
